# Shaping success: clinical implementation of a 3D-printed electron cutout program in external beam radiation therapy

**DOI:** 10.3389/fonc.2023.1237037

**Published:** 2023-08-09

**Authors:** Joseph B. Schulz, Clinton Gibson, Piotr Dubrowski, Caroline M. Marquez, Lynn Million, Yushen Qian, Lawrie Skinner, Amy S. Yu

**Affiliations:** Department of Radiation Oncology, Stanford University School of Medicine, Stanford, CA, United States

**Keywords:** 3D-printing, radiation therapy, electron, custom, tungsten BB, cutout

## Abstract

**Purpose:**

The integration of 3D-printing technology into radiation therapy (RT) has allowed for a novel method to develop personalized electron field-shaping blocks with improved accuracy. By obviating the need for handling highly toxic Cerrobend molds, the clinical workflow is significantly streamlined. This study aims to expound upon the clinical workflow of 3D-printed electron cutouts in RT and furnish one year of *in-vivo* dosimetry data.

**Methods and materials:**

3D-printed electron cutouts for 6x6 cm, 10x10 cm, and 15x15 cm electron applicators were designed and implemented into the clinical workflow after dosimetric commissioning to ensure congruence with the Cerrobend cutouts. The clinical workflow consisted of four parts: i) the cutout aperture was extracted from the treatment planning system (TPS). A 3D printable cutout was then generated automatically through custom scripts; ii) the cutout was 3D-printed with PLA filament, filled with tungsten ball bearings, and underwent quality assurance (QA) to verify density and dosimetry; iii) *in-vivo* dosimetry was performed with optically stimulated luminescence dosimeters (OSLDs) for a patient’s first treatment and compared to the calculated dose in the TPS; iv) after treatment completion, the 3D-printed cutout was recycled.

**Results:**

QA and *in-vivo* OSLD measurements were conducted (n=40). The electron cutouts produced were 6x6 cm (n=3), 10x10 cm (n=30), and 15x15 cm (n=7). The expected weight of the cutouts differed from the measured weight by 0.4 + 1.1%. The skin dose measured with the OSLDs was compared to the skin dose in the TPS on the central axis. The difference between the measured and TPS doses was 4.0 + 5.2%.

**Conclusion:**

The successful clinical implementation of 3D-printed cutouts reduced labor, costs, and removed the use of toxic materials in the workplace while meeting clinical dosimetric standards.

## 1 Introduction

The innovations in implementations of custom field shaping blocks for electron radiation therapy (RT) have remained stagnant since the introduction of the Cerrobend system in 1973 (1). Cerrobend, a low melting point alloy composed of bismuth, lead, tin, and cadmium, has been widely used despite its toxic composition. Efforts have been made to reduce the toxicity of Cerrobend through the availability of a commercially available cadmium-free composition. However, the overall toxic composition has resulted in strict regulatory requirements and expensive infrastructure (2, 3). For example, per the Occupational Safety and Health Administration (OSHA), a medical surveillance and examination program of blood testing of an employee’s cadmium and lead exposure must be upheld and enforced (4). Failure to do so can lead to significant financial penalties. A block cutting room must also be established with proper ventilation systems and personal protection equipment for safe and efficient production of the custom field shaping Cerrobend blocks. The laborious manual production process and associated uncertainties in aperture shape and placement have further highlighted the need for novel technologies to modernize the process.

3D-printing has been rapidly adopted in RT (5, 6) due to its ability to produce complex, patient-specific devices such as bolus (7) and developing QA devices to improve treatment safety (8, 9). Interest has also been garnered into the collimation of electron-based RT, due to the labor-intensive processes involved (10, 11). We aim to further the utilization of 3D-printing in RT by reporting on one year of clinical use of patient-specific 3D-printed electron cutouts within a large multi-center radiation oncology network. The use of a rigid shell, patient-specific aperture, and tungsten ball bearings improve treatment accuracy, optimize workflow, and reduce the handling of toxic materials.

We build on previous work proposing a novel methodology for tungsten-filled 3D-printed electron collimators for 6x6 cm and 10x10 cm cones, and the possibility for clinical implementation (12, 13). In this work, we outline i) the clinical commissioning of a novel 3D-printed electron cutout design for 15x15 cm electron cones; ii) the clinical implementation and workflow of the 15x15 cm, 10x10 cm, and 6x6 cm electron cutouts; iii) recycling and sustainability efforts; and iv) a year of *in-vivo* measurement data from clinical use. This study is the first clinical experience report in the effort to remove Cerrobend from a radiation oncology clinic. As part of our responsibility, we will also disseminate our clinical expertise on how to effectively implement the 3D-printed cutouts across various locations.

## 2 Methods and materials

### 2.1 15x15 cm 3D-printed electron cutout design and manufacture

**Figure 1:**
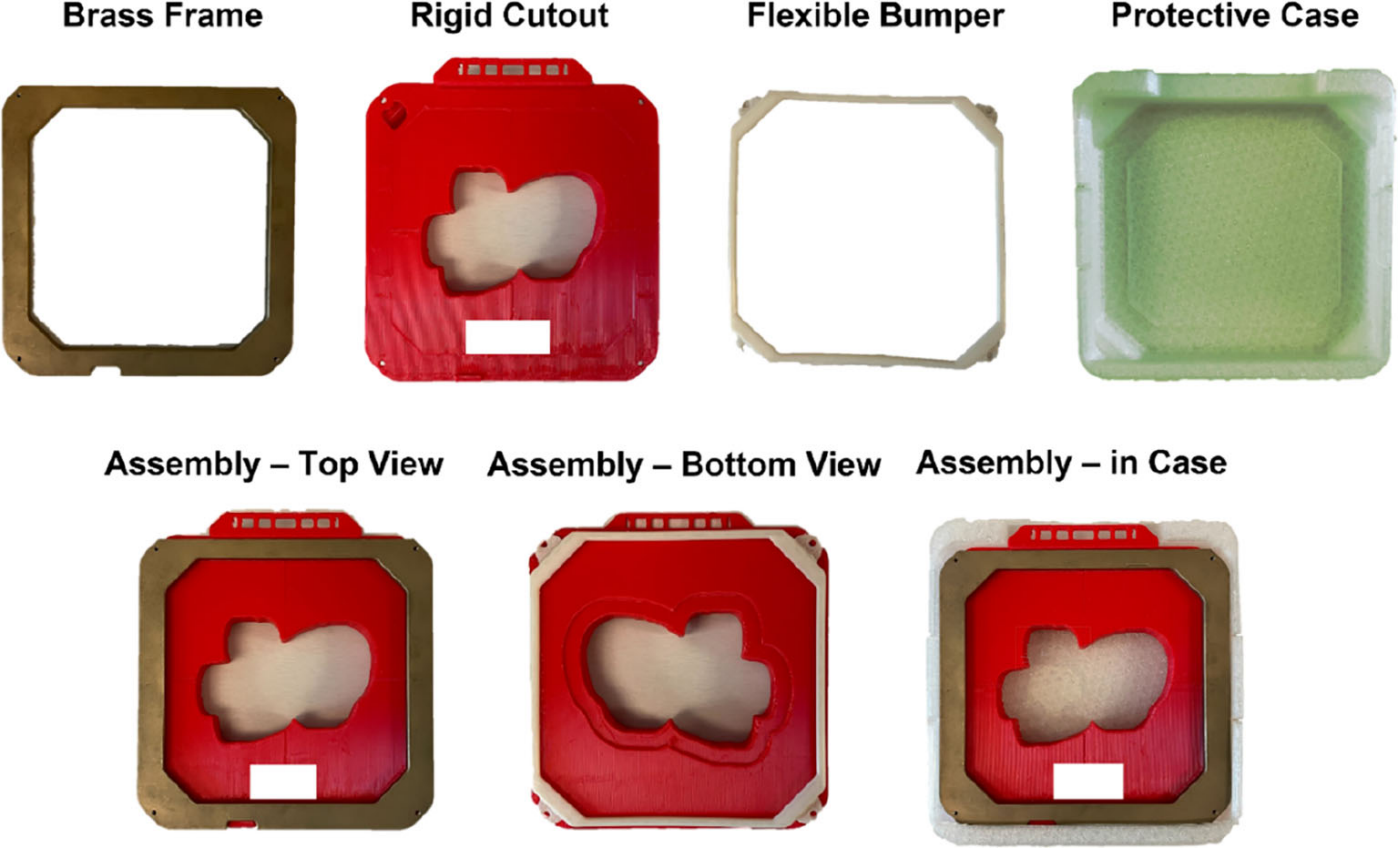
All fabricated components used in the assembly of the 15x15 cm 3D-printed electron cutout: a brass frame, the rigid 3D-printed PLA cutout, a flexible TPU bumper, and a flexible TPU protective case. The complete assembly is also included. All components are reusable or recyclable. Patient identifiers are redacted.

The novel 3D-printed 15x15 cm electron cutout was developed. It was composed of five main components: a PLA plastic cutout template (Tough PLA Filament, UltiMaker, Utrecht, Netherlands), a brass alloy frame, a 3D-printed TPU protective carrying case, a 3D-printed TPU flexible bumper (TPU 95A, UltiMaker) and tungsten ball bearings (BBs) (Tungsten Shot, Midwest Tungsten Service, Willowbrook, IL). The process of assembling these components are illustrated in **Figure 1** and viewable in the linked video (14).

**Figure 2:**
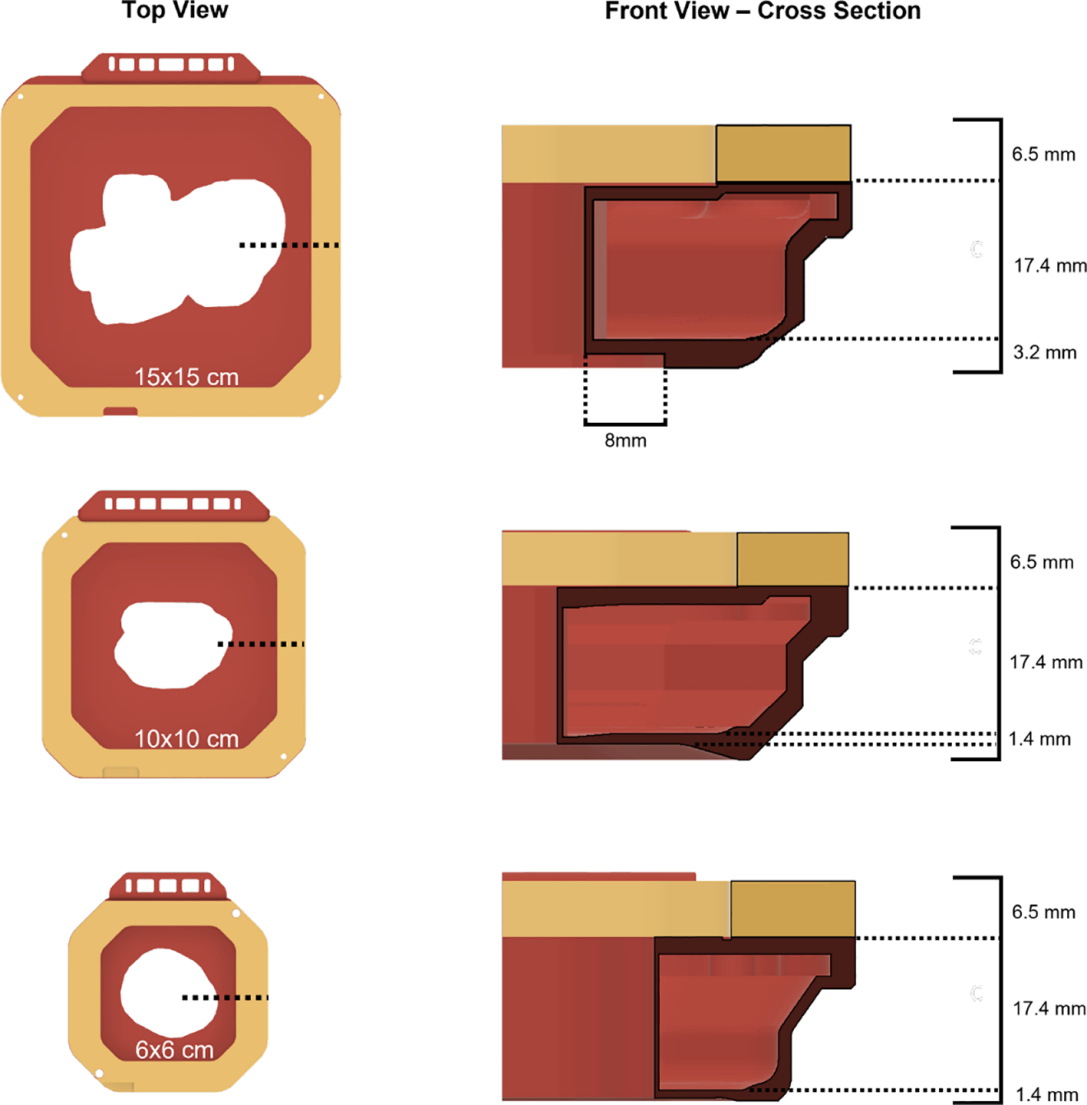
Top view and cross-sectional front views of all available 3D-printed electron cutout iterations: 15x15 cm, 10x10 cm, and 6x6 cm. The wall thickness for each cutout is 0.9 mm, 0.4 mm, and 0.4 mm respectively.

The new 15x15 cm template iteration was fundamentally a scaled-up version of the previous 6x6 cm and 10x10 cm electron cutout design, incorporating multiple technical considerations (13) (**Figure 2**). To accommodate the greater BB mass needed to properly fill this enlarged cutout, the interior wall thickness had been augmented to 0.9 mm (up from 0.4 mm in 6x6 cm and 10x10 cm versions), and the baseplate thickness had been extended by an additional 1.8 mm throughout the whole baseplate (as opposed to only along the perimeter 10x10 cm cutout design). However, this increase in thickness was specifically designed to be outside an 8 mm margin around the cutout wall, to preserve the cross-sectional thickness established in the earlier cutout designs (12).

Anonymized 15x15 cm clinical cutouts were 3D-printed to be compared to Cerrobend clinical cutouts used in completed treatments. Workflow improvements have been developed, utilizing scripting capabilities via the Eclipse Application Programming Interface (ESAPI) (Varian, Palo Alto, CA, USA) and the Python programming language. Clinical electron cutouts were automatically exported from Eclipse using an in-house script to produce a stereolithography (.stl) file ready for 3D-printing. The.stl file is then imported into UltiMaker Cura for slicing and 3D-printed via an UltiMaker S5. After printing, the 3D-printed cutouts were filled with 1.5 mm to 2 mm diameter tungsten BBs.

### 2.2 15x15 cm 3D-printed electron cutout commissioning dosimetry measurements

**Figure 3:**
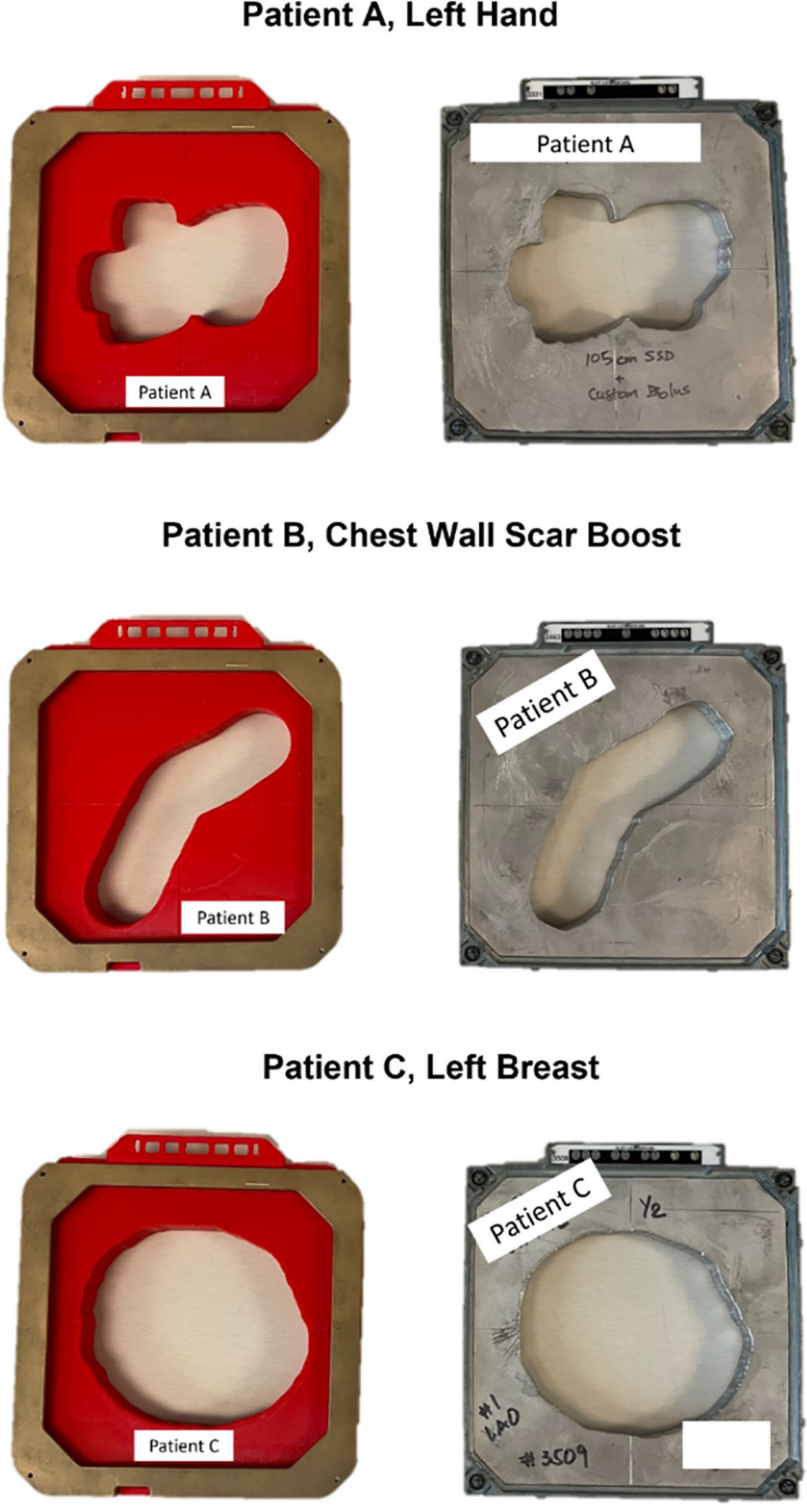
A top down view of the fully assembled 15x15 cm 3D-printed cutouts for each patient, positioned beside the clinically used Cerrobend cutouts. Patient identifiers were redacted.

Three distinct types of patient electron apertures had been chosen for this investigation, selected based on their unique field shapes and the associated disease sites: an irregularly shaped aperture used for a hand (patient A), a long, thin aperture used for a chest wall scar (patient B), and a nearly circular aperture used for a breast boost (patient C) (**Figure 3**). Dosimetric parameters that held clinical relevance for the 15x15 cm 3D-printed electron apertures were thoroughly examined. For each 3D-printed cutout, measurements of dose output, field shape, geometry, and surface dose were taken, and then each was compared to its corresponding Cerrobend cutout and to the treatment plan from the TPS.

#### 2.2.1 Dose measurements

Dose output measurements were performed in solid water with a pinpoint ionization chamber (PTW PinPoint chamber, PTW-Frieburg, Breisgau, Germany). The chamber was placed on central axis with 5 cm of backscatter at depths of 1.3 cm, 2.0 cm, 2.8 cm, 3.2 cm, and 2.3 cm, respectively, for the electron beam energies of 6, 9, 12, 16, 20 MeV (approximate values of *d_max_*) at 105 cm source to surface distance (SSD). For all output measurements, 400 Monitor Units (MUs) were delivered. Additionally, reference condition measurements were made at each energy using the standard 15x15 cm field size to establish daily linac output. Using the Eclipse TPS, the dose for each respective energy and depth was calculated at the central axis (CAX) for the patient-specific aperture at 105 cm SSD, as well as an open 15x15 cm field at 100 cm SSD. The ratio of the TPS doses were calculated and compared to the ratio of the 3D cutouts and Cerrobend measurements. Surface dose measurements were performed using a solid water phantom and a parallel plate ionization chamber (PTW Exradin A10, PTW-Frieburg, Breisgau, Germany), placed on the central axis, with 5 cm backscatter. The ratio of the readings between no buildup and the respective *d_max_* was then calculated.

#### 2.2.2 Light field aperture comparison

A detailed analysis was conducted to compare the light field apertures of three different elements: a 3D-printed cutout, a Cerrobend cutout, and a Treatment Planning System (TPS). To accomplish this, we projected the light field through each cutout while it was positioned at 100 cm from the source to surface distance (SSD), and traced the projection meticulously. In the case of the Cerrobend cutout, its block shape was directly exported from the TPS for the comparison. However, for the 3D-printed cutout, we adjusted the exported block shape by geometrically reducing it by 0.9 mm to account for the material of the wall. Following this, we digitized the resulting physical images and processed them for analysis with MATLAB (MathWorks located in Natick, MA, USA). We completed the analysis by first setting a threshold on the images, and then calculating the Jaccard distance. This was achieved by applying the built-in function. The Jaccard distance is used to measure the dissimilarity of sample sets and is described in Equation 1, where A and B are two sample sets. In the context of this work, A is either the 3D-printed cutout (3D) or the Cerrobend aperture (CB), and B is the TPS aperture; delineated respectively as *d_j_* (3*D*,*TPS*) and *d_j_* (*CB*,*TPS*).


(1) $$d_{j}\left(A,B\right)=\frac{\left|\left(A\cup^{\;}B\right)\right|-\left|\left(A\cap^{\;}B\right)\right|}{\left|\left(A\cup^{\;}B\right)\right|}$$

#### 2.2.3 Out-of-field transmission measurements

Transmission measurements were completed using GAFchromic film (EBT3-XD, Ashland, Wilmington, DE) placed on 5 cm of solid water for a single 3D-printed patient cutout and the Cerrobend counterpart. The cutouts were placed in the 15x15 cm electron applicator and 5000 MUs of 20 MeV were delivered. The film measurements taken for the Cerrobend cutout and 3D-printed cutout were overlayed in the software. Three regions of interest 4 cm x 2 cm in size were then inspected for radiation transmission via Film QA Pro (Ashland).

### 2.3 Clinical workflow for 3D-printed electron cutouts

In our previous work, a proposed clinical workflow was given for the 3D-printed cutouts ((13), Section 4.2). Overall, the workflow has been largely automated through custom scripting, removing the need for any manual computer-aided design (CAD) by the user for all cutout sizes. The initial manufacturing of all cutouts was similar to the procedure written for 15x15 cm cutouts in the previous section. The cutout verification and QA also remained largely unchanged from previous work (13). The empty 3D-printed shell was weighed first, filled with tightly packed tungsten BBs, weighed again, and compared to the calculated expected weight. The filling of shell with tungsten BBs was completed by holding the cutout at approximate 45-degree angle, gently shaking, and pouring the BBs until roughly level with the infill hole. The process varied by cutout size and required approximately 5-10 minutes. In addition, the cutout aperture square equivalent area was automatically calculated. If the area was less than a 4x4 cm square, pre-treatment output verification was performed with a pinpoint ion chamber and compared to the TPS. Instructional internal reference videos were produced, covering the use of the cutout generation application and the pre-treatment weight QA of the cutouts (14, 15).

### 2.4 *In vivo* dosimetry measurements

*In vivo* dosimetry was performed for the patient’s first treatment using nanoDots (LANDAUER, Glenwood, IL, USA) OSLDs placed at the central-axis (CAX) of the electron aperture light field on the patient’s skin. The measured OSLD dose was then compared to the approximate dose point on the patient’s body contour and recorded. The dose point measurements in the TPS were taken 0.8mm below the surface of the patient’s body contour to account for the noted inherent buildup of the OSLDs (16).

### 2.5 Recycling and sustainability efforts

At the end of treatment 3D-printed parts were recycled back into filament. This was done through Filabot’s array of products aimed at recycling plastic into materials for additive manufacturing (Filabot, Barre, VT, USA). The consumer-grade recycling system consisted of i) a plastic shredder to granulate discarded 3D-prints; ii) a heated extruder to melt the material into filament; iii) a fan-cooled path for rapid cooling and solidification; iv) a spooling rig; and v) a pelletizer to cut the filament into more regulated pellets as input material for step ii). The amount of material was weighed before and after the recycled filament extrusion, and data on filament diameter over time was collected.

## 3 Results

### 3.1 15x15 cm 3D-printed electron cutout commissioning dosimetry data

**Figure 4:**
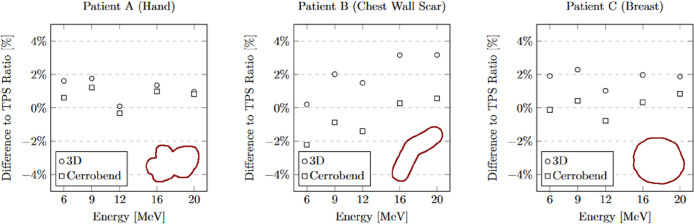
The comparison of 3D-Printed and Cerrobend output ratio to treatment planning system ratio. The apertures are shown in the lower right of each plot. The standard error for all measurements is within 0.15%.

The measurements of relative output dosimetry for the three patient-specific cutouts are depicted in **Figure 4**. Both the output from the 3D-printed cutouts and the Cerrobend cutouts aligned, on average, within 2% of the dose calculated by the TPS. The average output dose from the 3D-printed cutouts exceeded its Cerrobend counterparts by 1.63% when both were compared to the TPS-calculated dose. The largest deviation observed from the TPS-calculated dose in the case of the 3D-printed cutouts was 3.16% at 20 MeV for patient B, while for the Cerrobend cutouts, it was 2.21% at 6 MeV, again for patient B.

**Figure 5:**
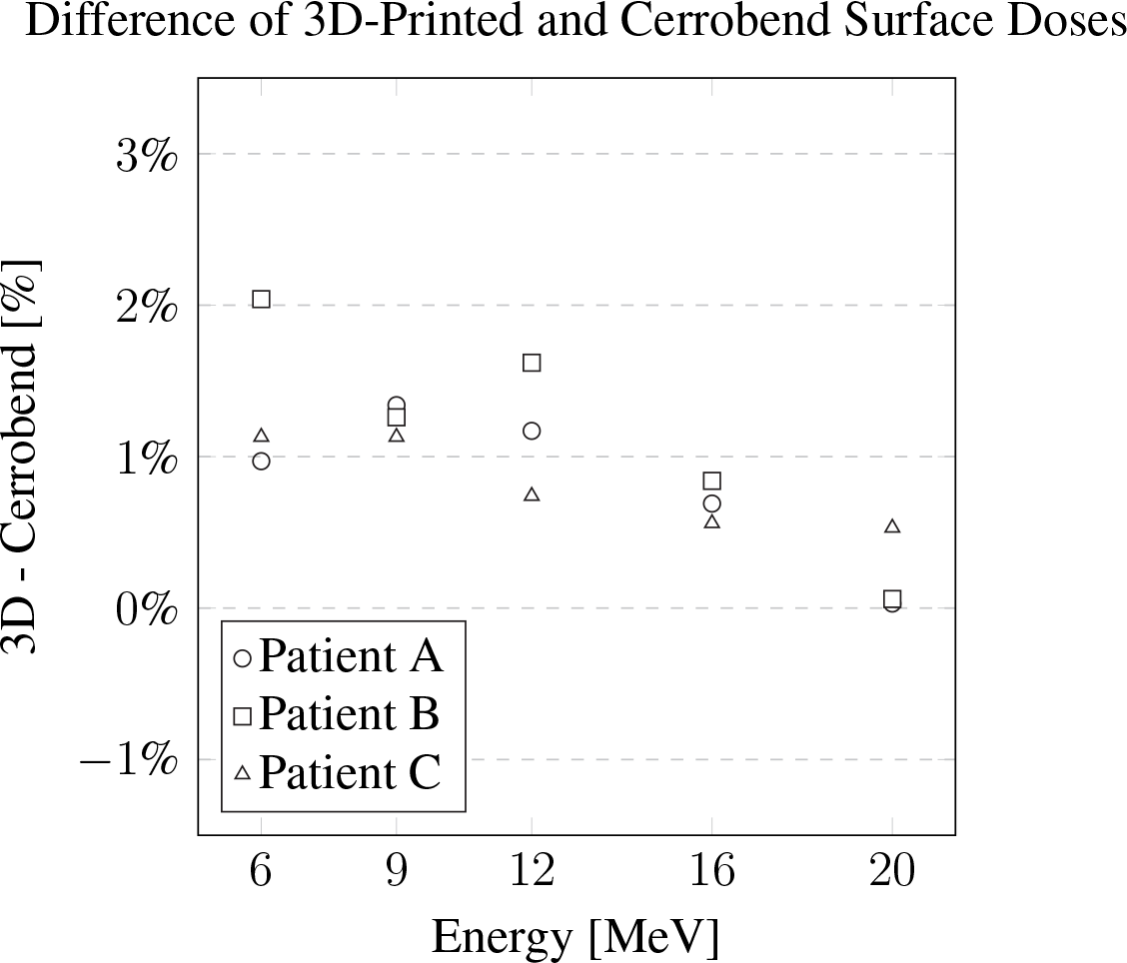
The difference of the 3D-printed electron cutouts surface dose measurements to the Cerrobend cutouts counterparts, relative to dose in the treatment planning system. The standard error for all measurements is within 0.1%.

The measurements of relative surface dose are presented in **Figure 5**. On average, the surface dose for the 3D-printed cutouts was found to be less than 1% higher when compared to their Cerrobend counterparts. The most notable deviation was seen in the case of patient B at 6 MeV, where the surface dose of the 3D-printed cutout exceeded its Cerrobend equivalent by 2.04%.

**Figure 6:**
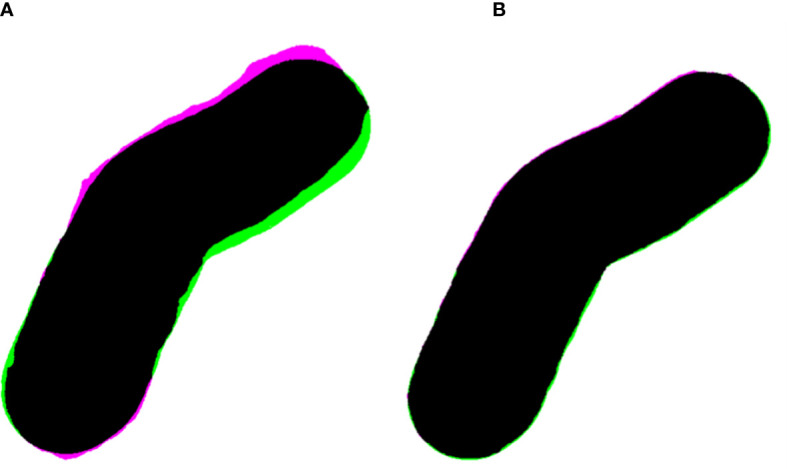
**(A)** Overlayed apertures (black) of the Cerrobend electron cutout (pink) and treatment planning system (TPS, green) at 100 cm SSD, computing a Jaccard distance of *d_j_* (*CB*,*TPS*) = 0.032. **(B)** Overlayed apertures (black) of the 3D-printed electron cutout (pink) and TPS (green) at 100 cm source to surface distance, computing a Jaccard distance of *d_j_* (3*D*,*TPS*) = 0.007.

The light field aperture comparison at 105 cm SSD between a manually made Cerrobend cutout and TPS aperture yielded a Jaccard distance of *d_j_* (*CB*,*TPS*) = 0.032. Comparison between the 3D-printed cutout and the TPS aperture yielded a Jaccard distance of *d_j_* (3*D*,*TPS*) = 0.007. The apertures and alignment are visualized in **Figure 6**, and the original cutouts are shown in **Figure 3**, patient B.

**Figure 7:**
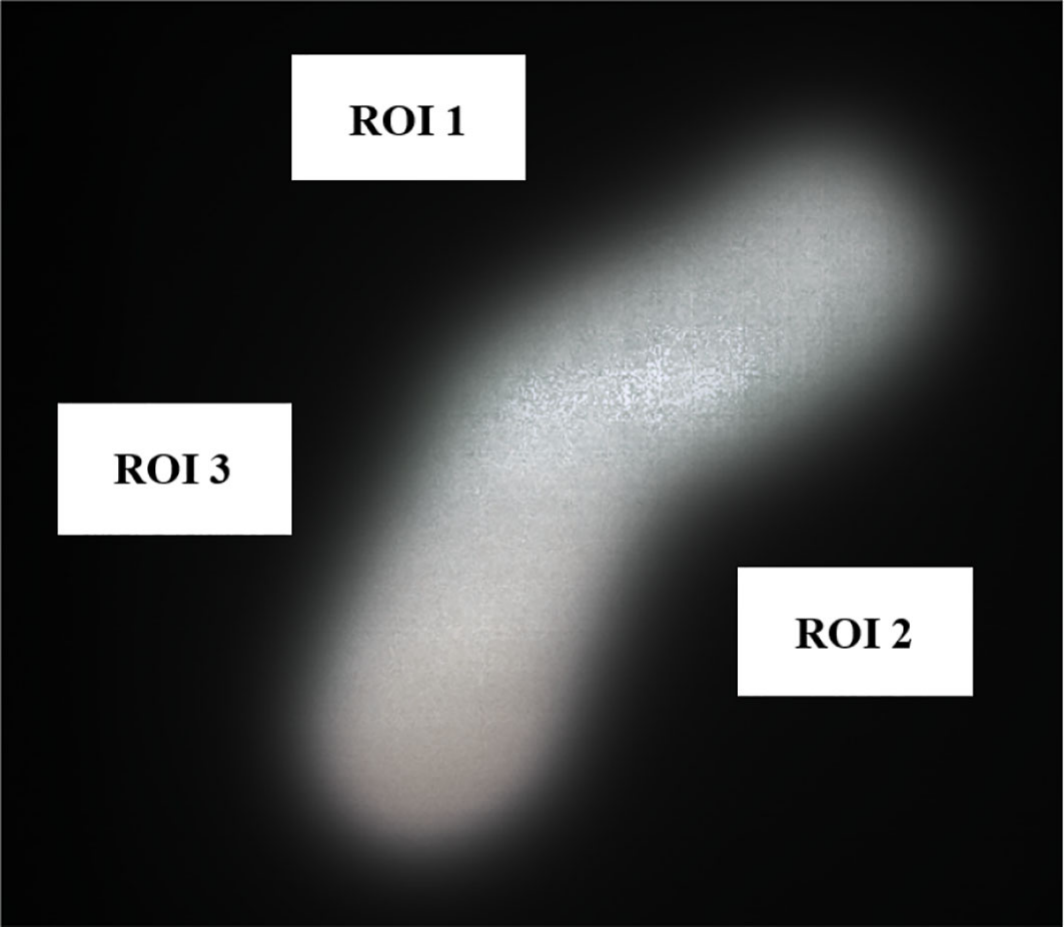
Overlayed film of 3D-printed and Cerrobend electron cutouts for patient B, displaying the 4 cm x 2 cm in size region of interests that were sampled.

On average for out-of-field transmission, the Cerrobend cutout was within error compared to its 3D-printed electron cutout counterpart. The locations of the regions of interest, which served as the basis for these measurements are illustrated in **Figure 7**. The statistics for each region of interest of the overlayed films are described in **Table 1**.

**Table 1:** Normalized dose region of interest measurements from the outside of the radiation field.

Cutout Type	ROI 1 [%]	ROI 2 [%]	ROI 3 [%]
Cerrobend	3.7 ± 0.5%	4.1 ± 0.5%	3.7 ± 0.8%
3D-printed	3.4 ± 0.6%	3.7 ± 0.6%	3.3 ± 0.8%

### 3.2 *In vivo* dosimetry data and cutout QA

**Figure 8:**
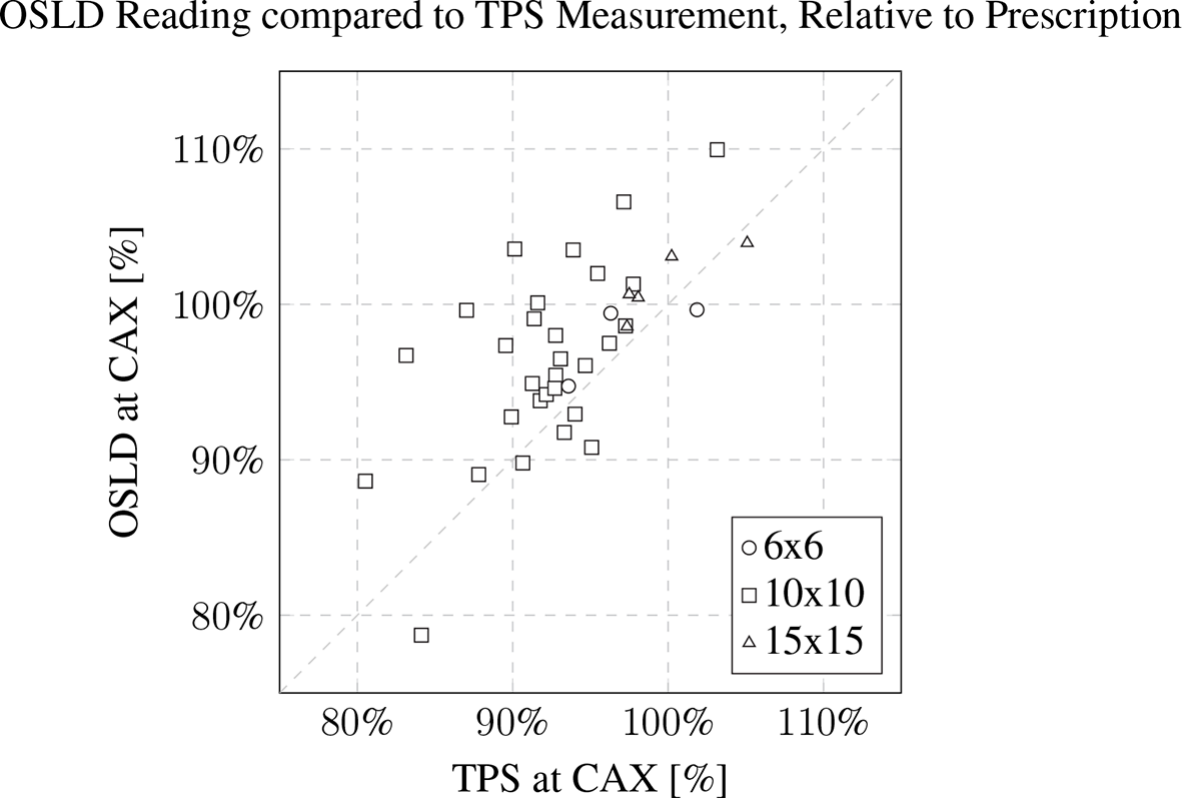
OSLD reading compared to TPS CAX measurement, relative to prescription. The patient’s 3D-printed electron cutout size is noted in the inset legend. The OSLDs used for measurement were screened variants with a manufacturer provided 5% measurement error.

Measurements were conducted for n=40 patients. The majority of the electron cutouts produced were the 10x10 cm iteration (n=30), with the remaining iterations of 15x15 cm (n=7) and 6x6 cm (n=3). For the 6x6, 10x10, and 15x15 electron cutouts respectively, the expected weight of the cutout differed from the measured weight by 1.5 ± 0.2%, 0.5 ± 1.0%, and -0.4 ± 0.9%. When compared to the prescribed dose fractionation, the measured dose deviated by 0.8 ± 2.7%, 4.7 ± 5.6%, and 1.7 ± 1.7%. This data is reported in **Table 2**. The OSLD measurements compared to the TPS are visualized in **Figure 8**.

**Table 2:** *In vivo* measurement statistics and cutout quality assurance.

Cutout Size, n	Mean difference to expected weight of cutouts ± SD [%]	Mean delivered dose compared to TPS ± SD [%]
6x6 (n = 3)	1.5 ± 0.2%	100.8 ± 2.7%
10x10 (n = 30)	0.5 ± 1.0%	104.7 ± 5.6%
15x15 (n = 7)	-0.4 ± 0.9%	101.7 ± 1.7%

### 3.3 Recycling efforts data

All PLA prints were recycled with an overall efficiency of 79 ± 12% between color batch runs. The spooling of filament averaged about 45 minutes per spool. The mean mass of all spools was 475 ± 145g. The best achieved filament diameter was 2.85 ± 0.09 mm.

## 4 Discussion

### 4.1 15x15 cm 3D-printed electron cutout commissioning dosimetry

The relative dose output measurements detailed a slight increase in dose for the 3D-printed cutouts, especially for the shape of patient B. The increase was discussed with our team of radiation oncologists and accepted. The increase is hypothesized to be due to the change in scattering, due the addition of the plastic walls in the aperture as previously observed (13). Surface dose measurements were slightly higher than Cerrobend cutouts, except for patient B. For the latter, we believe the issue might be the same as for the dose output measurements, where a small field aperture leads to a higher dose to the surface and central axis. The dissimilarity of the 3D-printed cutouts aperture and the Cerrobend aperture to the TPS aperture is illustrated through the light field aperture comparison. The ratio *R*, of the dissimilarities of the Cerrobend cutout to the TPS [*d_j_* (*CB*,*TPS*)] against the 3D-printed cutout to the TPS [*d_j_* (3*D*,*TPS*)] is calculated to be 457% as described in Equation 2. This is due to errors in the edge position and centering of the Cerrobend cutout, brought on by the more intensive manual labor involved in its production. Although not included in this report, previous penumbra and profile measurements have shown similar agreement with the TPS (12, 13).The out-of-field transmission was found to be lesser in the 3D-printed cutouts and is attributed to the greater baseplate thickness required to support the weight of the tungsten BBs.


(2) $$R=\frac{d_{j}\left(CB,TPS\right)}{d_{j}\left(3D,TPS\right)}$$

### 4.2 *In vivo* dosimetry and cutout QA

*In vivo* measurements exhibited a dose deviation of about 4% on average from the TPS. This deviation is inclusive of multiple factors. The OSLDs were screened variants, which establishes a 5% run-to-run variance by the manufacturer. The limitations of the Electron Monte Carlo dose calculation algorithm were also noted at the surface of the patient’s body contour.

The weight QA showcased the importance of an accurate establishment of expected mass of the cutouts to ensure complete packing of the tungsten BBs. The 1.7% greater measured mass of the 6x6 cm cutouts brings to attention the need for re-baselining of the expected weight, with the procedure established in our previous work (13).

### 4.3 Clinical implementation and workflow

The integration of 3D-printed cutouts into clinical workflows is generally straightforward due to less intensive regulation and minimal infrastructure requirements compared to the use of Cerrobend. 3D-printed electron cutouts reduced manual labor by an estimated 50% per cutout, from an approximate 40 minutes to 20 minutes per cutout, as reported by the practicing block technician. Examining all the patient electron blocks, the expansion of the cutouts to the 15x15 cm size presented here represents 16% of our clinical cases, and the three sizes 6x6 cm, 10x10 cm, 15x15 cm cover approximately 91% of our clinical cases. The remaining 4 cases are 20x20 cm cutouts.

The maintenance and uptime of a 3D-printer is greatly dependent on the specific make and model. We have found that our model, UltiMaker S5, required minimal upkeep. Occasionally, minor issues such as automatic bed-leveling failures and filament breakages do occur but are easily resolved using well-known and timely solutions. These maintenance tasks are typically handled by our physics assistants or by other experienced 3D-printing users, including our physicists. Production of incorrect 3D-printed cutouts are infrequent, and are mainly attributed to printer issues identified during the printing process. The failed 3D-prints and used 3D-printed devices that are not in contact with the patient are recycled and used only for prototyping novel designs or day-to-day designs to enhance our clinic. We only use vendor-purchased materials for patient-specific devices, such as the 3D-printed electron cutout, due the higher quality compared to our in-house filament.

The production of 3D-printed cutouts promotes sustainability, as all components of the electron cutouts are either reusable or recyclable. The 3D-printing of boluses has been previously established and is commonly used in clinical practice (7, 17, 18), and similar 3D-printing programs and workflows can be used for the production of electron cutouts. The only additional purchase required is the rigid brass frames and the tungsten BBs. The workflow for the production of 3D-printed cutouts is similar to previous work, with the addition of automated scripts via the ESAPI and Python programming language. The utilization of multiple 3D-printers can allow for the simultaneous production of electron cutouts in the same space used to produce electron cutouts sequentially in a block shop. Overall, the low footprint and ease of use of the 3D-printers support cutout scalability within larger hospital networks and their accessibility for smaller clinics.

### 4.4 Multi-site rollout

Over the course of a year, we rolled out the production of 3D-printed cutouts to two other institutional locations for their own patient populations. This process was initiated through first identifying a 3D-printer model that met our requirements of i) remote printing capabilities, ii) minimal maintenance, and iii) printer cost. By utilizing the remote printing capability of the 3D-printers, production workload can be distributed between these locations to increase the overall production capacity. For example, a technician situated at an institutional clinic with a smaller patient population is able to remotely initiate the 3D-printing process for a 3D-printer housed at a larger institutional clinic, and vice-versa. The ability for cross-coverage greatly enhances the efficiency of production and allocation of resources. By purchasing and deploying the same model of 3D-printer at each institutional location, we are able to use one printing profile for all printers.

We trained one individual at each institutional location as a super-user. This training entailed an in-person dry run for the full workflow of a 3D-printed electron cutout, and then an actual patient case. Many materials and information to maintain the program, such as the software, printing profiles, instructional documents, instructional videos, and Internet Protocol (IP) addresses of the 3D-printers are provided on a designated web page on our internal institutional website.

This cross-coverage approach allows for better load balancing between facilities, ensuring that resources are utilized efficiently while reducing the likelihood of bottlenecks in production. This is especially important as a major drawback of 3D-printing is its slowness. The 3D-printed cutout print time was approximately 4 hours for the 6x6 cm cutout, 5 hours for 10x10 cm cutout, and 7 hours for the 15x15 cm cutout. We are currently exploring the capabilities of the newer generation of 3D-printers, which can potentially reduce these printing times by half. Additionally, this distributed approach can lead to cost savings for individual institutions. By sharing the workload and resources, each institution can optimize the use of their existing 3D-printers without having to invest in multiple machines or additional personnel. Furthermore, this model allows for a more effective use of staff time, as they can focus on other clinical tasks instead of being burdened with manually manufacturing the electron cutouts.

### 4.5 Cost analysis

Cost analysis is a crucial aspect of decision-making in any organization. It is particularly critical when evaluating new technologies and equipment to determine if the benefits outweigh the costs. In this context, a cost analysis was completed which compared the 3D-printed method to the standard Cerrobend method in order to provide valuable insight. The approximate costs associated with each methodology are described in **Table 3**, with an example patient population of 50 electron cutouts produced over one year and further details noted in Appendix 6.1.

**Table 3:** Approximated total cost for maintaining a Cerrobend or a 3D-printed cutout program, for 50 electron cutouts per year.

	Upfront Cost (US$)	Yearly Operating Cost (US$)
Cerrobend Cutout Program		
Block Room Equipment	$30,000	–
50 lbs Cerrobend Alloy	$850	–
Shipping	–	$2,500
0.025 FTE	–	$1,500
Total:	$30,850	$4,000
3D Printed Cutout Program		
3D-Printer (6x6 cm, 10x10 cm, 15x15 cm)	$7,000	–
10 Rigid Brass Alloy Frames	$1,000	–
30 lbs Tungsten Ball Bearings	$3,450	–
External Manufactured Cutouts (20x20 cm)	–	$1,150
3D-Printing Filament	$210	$250
0.0125 FTE	–	$670
Total:	$11,600	$2,070

There are several limitations to this cost analysis that are not accounted for. For example, the cost of the physical space required at the clinic for a block room is not considered. If there is a high cost associated with the physical space needed, the 3D-printing program would be more appealing due to the minimal space requirement to house a 3D-printer, which is typically less than a square meter. Moreover, factors such as training, routine blood testing for heavy metals, maintenance, and equipment upgrades have not been included in the cost analysis. These additional costs could potentially impact the overall cost-effectiveness of each method.

While the upfront cost of the 3D-printed program is significantly lower than that of the Cerrobend program, institutions must consider other factors, such as space requirements, maintenance, and equipment upgrades, as well as the trade-offs between cost, efficiency, and quality. By taking all these factors into account, organizations can choose the most suitable method for their specific needs and patient population.

## 5 Conclusion

Overall, the adoption of 3D-printed cutouts into clinical practice has the potential to improve patient care while reducing costs and streamlining workflows. With the increasing availability and affordability of 3D-printing technology, it is likely that the use of 3D-printed cutouts will become more widespread in RT clinics and can provide for more precise and customizable treatment options. Additionally, the environmental benefits of using 3D-printed cutouts, such as the removal of toxic heavy metals and more efficient use of resources, make this technology an attractive option for clinics seeking to minimize their ecological footprint. By evaluating the costs and benefits of using 3D-printed cutouts, as well as the potential impact on patient care and clinical workflows, institutions can make informed decisions that lead to increased efficiency, and ultimately, better overall patient care.

## Data availability statement

The raw data supporting the conclusions of this article will be made available by the authors, without undue reservation.

## Author contributions

AY, LS, PD, JS, CG, LM, CM, and YQ contributed to the conception and design of the study. JS collected the data. JS, PD, AY, and LS performed the data analysis. JS wrote the first draft of the manuscript. All authors contributed to the article and approved the submitted version.

## Appendix

**Cost analysis**

Regarding the approximate costs of establishing and maintaining a Cerrobend Cutout program, we obtained a quote for a fully equipped Cerrobend block room, which totaled approximately $30,000. Cerrobend alloy was only offered as a 50lbs unit for $850. To ship to satellite clinics that do not have a block room was estimated at $50 per cutout, for a total of $2,500. Our institution’s technicians with experience in Cerrobend electron cutout creation estimated that the manual labor involved in manufacturing an electron cutout was 40 minutes on average. Assuming a Full-Time Equivalent (FTE) of 40 hours per week and a rate of $45 per hour, a technician will spend 0.025 FTE over the course of a year to produce 50 Cerrobend electron cutouts, for an overall approximate labor cost of $1,500. In total, we approximate the upfront cost for the program would be $30,850, and the yearly operating cost would be $4,000.

Regarding the approximate costs of establishing and maintaining a 3D-printed electron cutout program, the 3D-printer model we utilize currently retails for $7,000. The material and labor costs for machining 10 reusable rigid brass alloy frames are approximately $100 per frame, for a total of $1,000. The cost of 30lbs of 2 mm diameter tungsten ball bearings is approximately $115 per pound, totaling $3,450. Since our program implementation only extends to 15x15 cutouts, some 20x20 and 25x25 cutouts may need external fabrication and is quoted at approximately $230 per cutout, with an estimated requirement for 5 out of 50 patients, totaling $1,150. Initially TPU filament is needed to produce five bumpers and protective carrying cases for the 6x6cm, 10x10cm, and 15x15cm cutout sizes; this is estimated to require approximately 15lbs of filament, equivalent to seven $30 1 kg spools, for a total of $210. The filament required over the course of the year to produce the 3D-printed electron cutouts is estimated to be $250, or 5 $50 1 kg tough PLA spools. This is calculated assuming 5 out of 50 patients require a 6x6cm cutout (50 g of material needed per cutout), 30 out of 50 patients require a 10x10 cm cutout (100 g of material needed per cutout), and 10out of 50 patients require a 15x15 cm cutout (150 g of material needed per cutout). Overall, this equates to 4.75 kg of material needed per year. Our institution’s technicians with experience in 3D-printed electron cutout creation estimated that the manual labor involved in manufacturing an electron cutout was 20 minutes on average. Assuming an FTE of 40 hours per week and a rate of $45 per hour, a technician will spend 0.0125 FTE over the course of a year to produce 45 3D-printed electron cutouts, for an overall approximate labor cost of $670. In total, we approximate the upfront cost for the 3D-printed electron cutout program to be $11,600, and the yearly operating cost to be $2,070.
